# More than just anemia: Uncovering celiac disease beneath the surface: A case report

**DOI:** 10.1002/jpr3.70251

**Published:** 2026-10-15

**Authors:** Evan Greenhall, Bri Best, David F. Carpenteri, Paula Prieto‐Tizzard

**Affiliations:** ^1^ Division of Pediatric Gastroenterology, Phoenix Children's Hospital Phoenix Arizona USA; ^2^ Department of Biorepository and Research Phoenix Children's Hospital Phoenix Arizona USA

**Keywords:** copper deficiency, malabsorption, micronutrient deficiency, normocytic anemia

## Abstract

Celiac disease (CD) is an immune‐mediated disorder triggered by gluten in genetically susceptible individuals. Although iron deficiency anemia is a common presentation, other micronutrient deficiencies may occur. We describe a 3.5‐year‐old female whose initial presentation of CD was severe, asymptomatic normocytic anemia secondary to copper (Cu) deficiency. She presented with mild abdominal symptoms, weight loss, and profound anemia (hemoglobin as low as 1.6 g/dL) requiring transfusions. Extensive infectious, inflammatory, hematologic, and oncologic evaluations were unrevealing. Workup demonstrated markedly elevated celiac serologies and low serum Cu. Endoscopy confirmed Marsh 3B duodenal changes consistent with CD. Treatment with a strict gluten‐free diet and Cu supplementation led to normalization of hemoglobin, Cu levels, and growth parameters. This case highlights Cu deficiency–associated anemia as a rare pediatric presentation of CD and underscores the importance of considering CD in severe unexplained anemia with micronutrient deficiencies.

## 1 INTRODUCTION

Celiac disease (CD) is an immune‐mediated systemic disorder triggered by gluten and related prolamin that occur in genetically susceptible individuals.^1^ The global prevalence of CD ranges from 0.7% to 1.4%.^1^ Multiple micronutrient deficiencies have been reported in patients with CD including iron, folate, zinc, B12, and copper.^2^ Iron deficiency anemia is a well‐known presentation of this disorder, but other causes of anemia can be seen.^3^ We present a pediatric patient with primary presentation of CD being asymptomatic severe normocytic anemia in the setting of copper (Cu) deficiency.

## 2 CASE REPORT

A previously healthy, fully immunized, 3.5‐year‐old female presented to her pediatrician with a 4‐month history of intermittent mild abdominal pain, anorexia for 4 weeks, and a 1.36 kg weight loss over 6 weeks. Body mass index: 13.9 (*Z*‐score = –1.55). She was referred to the emergency department for further evaluation.

Initial evaluation revealed severe normocytic anemia (hemoglobin [Hgb] 2.1 gm/dL; normal: 11.5–13.5 gm/dL), leukopenia (white blood cell 3.7 K/μL; normal: 6–17 K/μL), and thrombocytosis (Plt 511 K/μL; normal: 140–450 K/μL]. Chest radiograph and abdominal computed tomography were unremarkable.

On admission, she was tachycardic but normotensive and appeared pale with mild abdominal distention. Repeat serology showed Hgb 1.6 g/dL with a reticulocyte count of 3%. She required multiple transfusions to maintain her Hgb >7 g/dL. Additionally, she was noted to have elevated erythrocyte sedimentation rate of 101 mm/h (normal 0–10 mm/h) and hypoalbuminemia of 3.1 g/dL (normal 3.5–5.2 g/dL). Her C‐reactive protein and homocysteine levels were normal.

Further reassuring workup included, thyroid stimulating hormone, folate, cobalamin, iron panel, fecal occult blood, throat culture for group A Streptococcal, stool infection PCR, *Clostridium difficile* antigen and toxin, *Helicobacter pylori* stool antigen, Parvovirus B19, Ebstein Barr Virus, antinuclear antibody, double‐stranded DNA and C3/C4. Imaging studies that were negative included abdominal ultrasound and Meckel's scan.

Hematology team was consulted due to concerns of malignancy and extensive evaluation was done including hemoglobin electrophoresis, echocardiogram, and a bone marrow biopsy; all normal. Heavy metals were notable for normal zinc and low serum Cu of 27.2 μg/dL (normal: 75–153 μg/dL). On day 10 of hospitalization, fecal calprotectin returned elevated at 227 μg/g prompting gastroenterology consultation.

Further evaluation at that time revealed markedly elevated tissue transglutaminase (tTG) immunoglobulin A (IgA) of 128 U/mL (normal <15 U/mL), tTG immunoglobulin G (IgG) 116 U/mL (normal <7 U/mL), deaminated gliadin peptide (DGP) IgA >142 U/mL (normal <7 U/mL), DGP IgG >302 U/mL (normal <7 U/mL), and endomysial antibody IgA >1:2560 (normal <1:10). Esophagogastroduodenoscopy was notable for duodenal scalloping and nodularity (Figure 1), with histopathology confirming Marsh 3B duodenal changes (villous atrophy, crypt hyperplasia, >40 intraepithelial lymphocytes/100 epithelial cells), and lymphocytic gastritis, findings consistent with CD (Figures 2 and 3). The colon and terminal ileum were endoscopically and histologically unremarkable.

**Figure 1:**
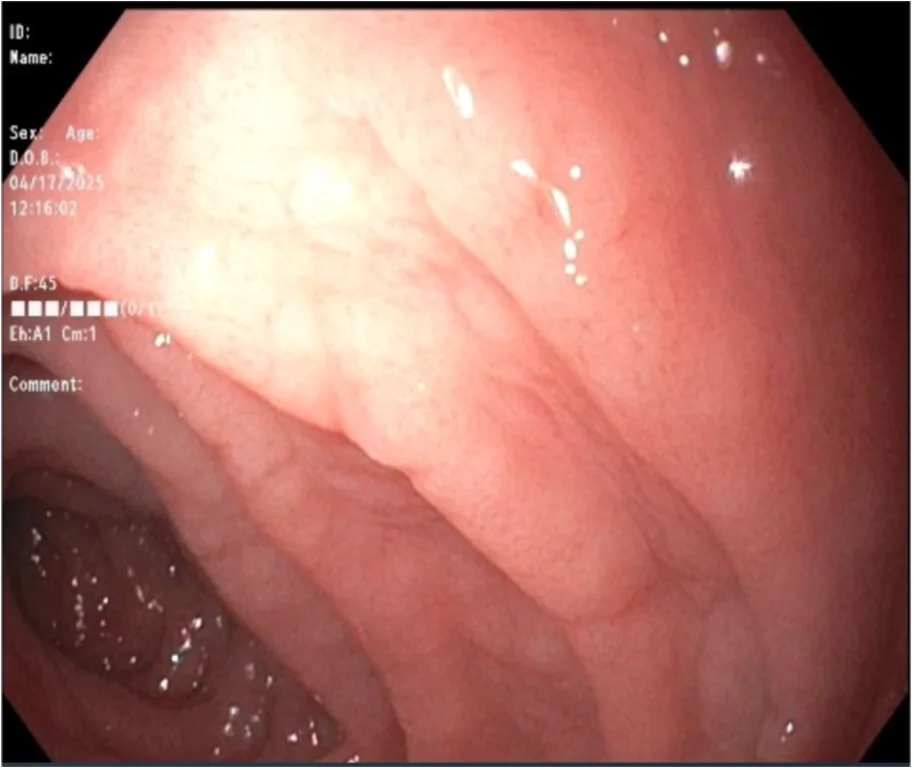
Endoscopic view of second portion of duodenum with scalloping and visual edema.

**Figure 2:**
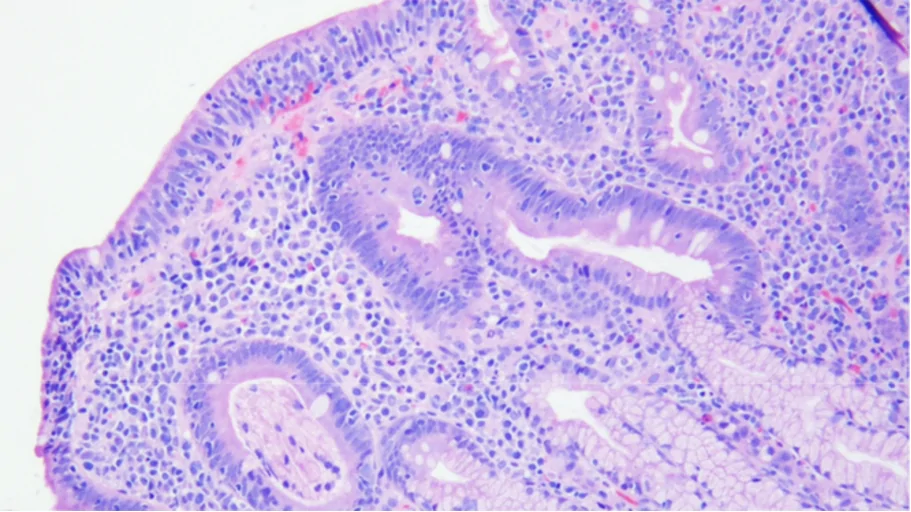
Hematoxylin and eosin ×10 duodenal mucosa with villous blunting, crypt hyperplasia, and intraepithelial lymphocytosis.

**Figure 3:**
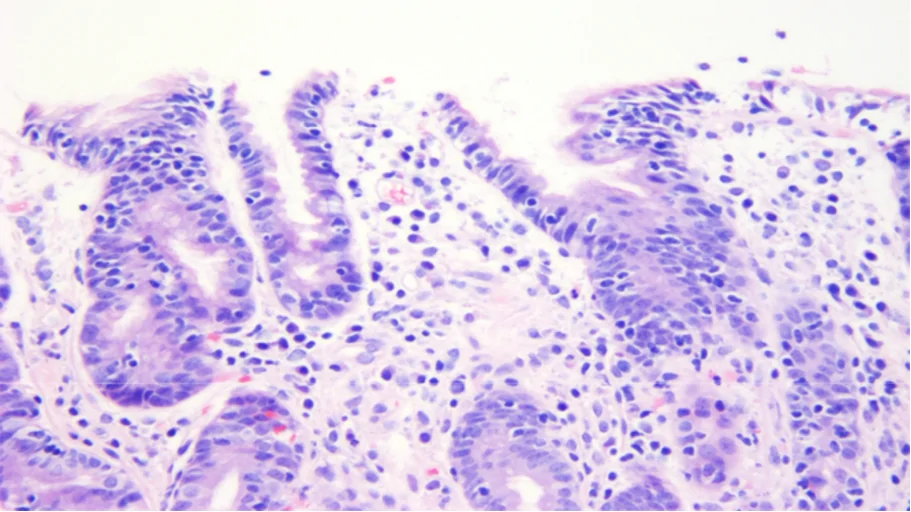
Hematoxylin and eosin ×20 gastric antrum with intraepithelial lymphocytosis.

The patient was initiated on a strict gluten‐free diet, supplementation of Cu Gluconate 2 mg/day and vitamin D, and received detailed nutritional counseling. She maintained Hgb >7 g/dL, with discharge Hgb 9.6 g/dL, and was discharged home in stable condition with outpatient hematology and gastroenterology follow‐up.

Since discharge, patient completed 90 days of Cu supplementation, has remained on strict gluten‐free diet with resultant normalization of anemia and serum Cu levels as well as improvement in growth trajectory. At the last visit, the patient is now without malnutrition and with a BMI of 15.5 (*Z*‐score = 0.19).

## 3 DISCUSSION

We describe a rare initial presentation of asymptomatic CD case characterized by severe normocytic anemia in the setting of Cu deficiency. Up to 69% of patients newly diagnosed with CD present with iron deficiency anemia.^3^ Although Cu deficiency is a recognized complication of malabsorptive syndromes, it is less commonly reported in CD, with prior reports limited to adults.^4^ Despite being less common than iron or zinc deficiency, Cu deficiency has been reported in approximately 6%–33% of patients with CD, with prevalence varying by cohort and diagnostic criteria, suggesting that it is a clinically relevant complication of CD.^4^

Cu deficiency has been observed in patients with chronic malabsorption due to upper gastrointestinal surgery, zinc overload, copper chelation, iron supplementation, dietary deficiency, and dependence on total parenteral nutrition. Cu is primarily absorbed in the stomach and proximal duodenum. Because CD‐associated inflammatory enteropathy predominantly affects the proximal small intestine, particularly the duodenum—a major site of copper absorption—mucosal injury in this region may impair copper uptake and predispose to copper deficiency.

It is an important component of ferroxidase, hephaestin, and ceruloplasmin, playing a key role in iron homeostasis; when deficient, it can lead to impaired hemoglobin synthesis.^5^ Other described presentations of Cu deficiency include myelopathy and optic neuropathy.^5^

Treatment of Cu deficiency in CD is not well described in the literature but few case reports document resolution of deficiency with gluten free diet.^2^, ^6^ In 2019, Altarelli et al. described an algorithm for treatment of Cu deficiency based on etiology and serum Cu level, but this did not describe replacement in CD. For patients with bariatric surgery induced deficiency, recommendation was for 3–8 mg/day of oral Cu sulfate or gluconate until resolution of Cu level.^2^ It was noted that hematologic manifestations should normalize by 12 weeks.^2^

CD presentation is variable and at times can be elusive. Although it usually presents with gastrointestinal symptoms, many times patients will present with extraintestinal manifestations or can remain asymptomatic for years.^7^ Extraintestinal symptoms include dermatitis herpetiformis, dental enamel hypoplasia of permanent teeth, osteopenia/osteoporosis, short stature, delayed puberty, and iron deficiency anemia.^7^

Treatment for CD is with strict gluten‐free diet. Although this will improve nutritional status some micronutrient deficiencies can still be seen such as iron, vitamin B12, folate, vitamin D, calcium, zinc, selenium, and magnesium.^8^ Monitoring for continued deficiencies should be considered.

Due to its varied presentation, it is recommended that testing for CD should be considered in those with less typical symptoms including constipation, linear growth failure, anemia, fatigue, arthralgia, and elevated liver enzymes.^3^ Therefore, in a patient with severe anemia and micronutrient deficiencies, CD should remain with high suspicion.

## 4 CONCLUSION

We present a novel case of normocytic anemia due to copper deficiency as the initial presentation of CD, highlighting the importance of recognizing the diverse manifestations of CD across primary and specialty care, as well as the value of assessing nutritional markers in complex diagnoses.

## CONFLICT OF INTEREST STATEMENT

The authors declare no conflicts of interest.

## ETHICS STATEMENT

This study was approved by the Phoenix Children's Institutional Review board (IRB): IRB‐25‐150. Informed consent was not obtained and waived by the IRB.

## Data Availability

Data sharing is not applicable to this article as no datasets were generated or analyzed during the current study.
